# Clinical, individual and environmental factors related to children’s health-related quality of life following treatment under general anaesthetic for dental caries: a path analysis

**DOI:** 10.1007/s40368-022-00695-w

**Published:** 2022-02-03

**Authors:** R. Knapp, Zoe Marshman, Fiona Gilchrist, Mario Vettore, Helen Rodd

**Affiliations:** 1 Academic Unit of Oral Health, Dentistry and Society, School of Clinical Dentistry, University of Sheffield, Sheffield, UK; 2 Department of Health and Nursing Sciences, University of Agder, Kristiansand, Norway

**Keywords:** Oral health, Quality of life, Paediatric dentistry, Caries

## Abstract

**Objective:**

To examine the impact of clinical, individual, and environmental factors on children’s oral health-related quality of life (OHRQoL) and overall health-related quality of life (HRQoL) following dental caries management under general anaesthetic (GA).

**Methods:**

Participants comprised 5- to 16-year-old children who were referred to a British Dental Hospital, for the management of their dental caries under GA. The Caries Impacts and Experiences Questionnaire for Children (CARIES-QC) and the Child Health Utility 9D (CHU9D) were used to assess child-reported OHRQoL and HRQoL, respectively, at baseline and 3-months follow up. A theoretical conceptual model, based on the Wilson and Cleary model of HRQOL, was evaluated using path analysis to explore indirect and direct relationships of the clinical, individual, and environmental variables on the quality of life outcomes following treatment.

**Results:**

85 children completed the study. Path analyses revealed that 47% of the variance in OHRQoL scores was accounted for by the variables in the model. There were significant relationships between change in OHRQoL score and treatment type [extraction only vs. combination care (*β* = 1.41, *p* = 0.07)] and number of extractions (*β* = 0.46, *p* < 0.001). A higher number of tooth extractions was associated with poorer OHRQoL and HRQoL following treatment.

**Conclusions:**

Treatment type, via number of extractions, may significantly impact on child OHRQoL and HRQoL following treatment under GA. However, to identify any other factors, that might affect these key outcomes, further enquiry is warranted with a bigger sample.

## Introduction

Dental caries affect around 573 million worldwide (Kassebaum et al. 2017) and, for some young patients, treatment under general anaesthetic (GA) remains a common treatment approach. Oral health-related quality of life (OHRQoL) measures have been increasingly used to evaluate the impact of oral diseases and the effect of dental treatment from a patient perspective. A systematic review of the impact of dental caries and its treatment under GA showed that child OHRQoL significantly improves following treatment, although some aspects of their quality-of-life worsened following treatment. In addition, the review showed that all the studies to date have simply reported change scores following treatment under GA and did not consider the effect of other variables on OHRQoL outcomes (Knapp et al. 2017).

More recently, theoretical models of health-related quality of life (HRQoL) have been applied to gain greater insight into which underlying factors may predict positive outcomes following health interventions. A well-known model, developed by Wilson and Cleary (1995) incorporates individual, environmental, and clinical factors which may potentially impact OHRQoL and has proven an appropriate approach to underpin previous dental intervention studies (Baker et al. 2007; Hasmun et al. 2020).

To date, several studies have investigated the overall impact of dental treatment under GA on children’s OHRQoL, but surprisingly little is known about the modifying effects of other clinical and environmental factors on these outcomes. For example, little is known about how a child’s deprivation status, symptoms, caries experience or required number of extractions may influence OHRQoL following treatment. Furthermore, previous dental GA studies have not incorporated a theoretical model of health to guide their analysis and explain their findings. Thus, the broad aim of this study was to determine which key clinical, individual, and environmental factors impact children’s OHRQoL and HRQoL following treatment for dental caries under GA according to the Wilson and Cleary theoretical model. Greater understanding of both direct and indirect effects of these variables has important implications for clinical practice and policy, for example, by identifying modifiable environmental, clinical or individual factors which could improve outcomes.

## Materials and methods

### Study design and study sample

Approval for this research, and a related study (Knapp et al. 2021), was obtained (NHS Research Ethics ID: 16/SS/0187). A convenience sample was drawn from children attending new patient assessment clinics at a British Dental Hospital over a 2-year period (January 2017 to January 2019). In the main, these participants were pre-cooperative younger patients with high caries experience, a history of dental infection, and who had been referred by their own dentist (or doctor) to a specialist paediatric dentistry service for a proposed dental GA. All patients were first assessed by senior paediatric dentistry and alternatives to GA were always considered where appropriate, along with the risk of GA, in line with UK National Clinical Guidelines (Association of Paediatric Anaesthetists of Great Britain & Ireland 2016). Following this assessment, the consultant enquired if the family would like to talk to one of the researchers (R.K) about the proposed study. Children were eligible for study inclusion if they met the following criteria: aged 5- to -16-years; presenting with dental caries that necessitated management under GA; no other dental conditions (such as trauma or enamel defects); medically fit and well; children and parents/guardians able to speak/read English. Families were given written and verbal information about the research project, time to reflect, and written consent (parents) and assent (child) was obtained prior to their participation. Data collection (described below) was at two time points: at the time of this initial assessment and at a routine review appointment 3-months following the dental GA.

A sample size calculation identified that a minimum of 75 child participants were required to explore the effects of variables in the model using path analysis, including 11 observed variables (power = 0.8, *α* = 0.05, detectable significant effects = 0.11).

### Path analysis

Path analysis (a form of multiple regression analysis) was undertaken to allow a more detailed understanding of any causal relationships existing between the different variables included in this investigation (Stage et al. 2004). Path analysis involves testing how well the observed data fits the proposed model, followed by the analysis of direct and indirect effects. This study followed guidelines proposed by Stage et al. (2004) whereby the model is tested for fit to the data before direct and indirect effects are analysed. Stages include identification of a full theoretical model to be tested; refining of the full model using the principle of parsimony; reporting of fit indices for the full and parsimonious final models; illustration of the parsimonious model, including direct and indirect effects.

### Theoretical model

The Wilson and Cleary model of HRQoL was used to underpin the research and to help identify which variables to include in the final path analysis (Wilson and Cleary 1995). This is a well-established theoretical model of OHRQoL which essentially comprises five components: the individual’s symptoms; functions, perceptions of general health, biological/physiological factors and overall self-reported overall QoL. Individual and environmental factors are also included in the overall model.

**Fig. 1:**
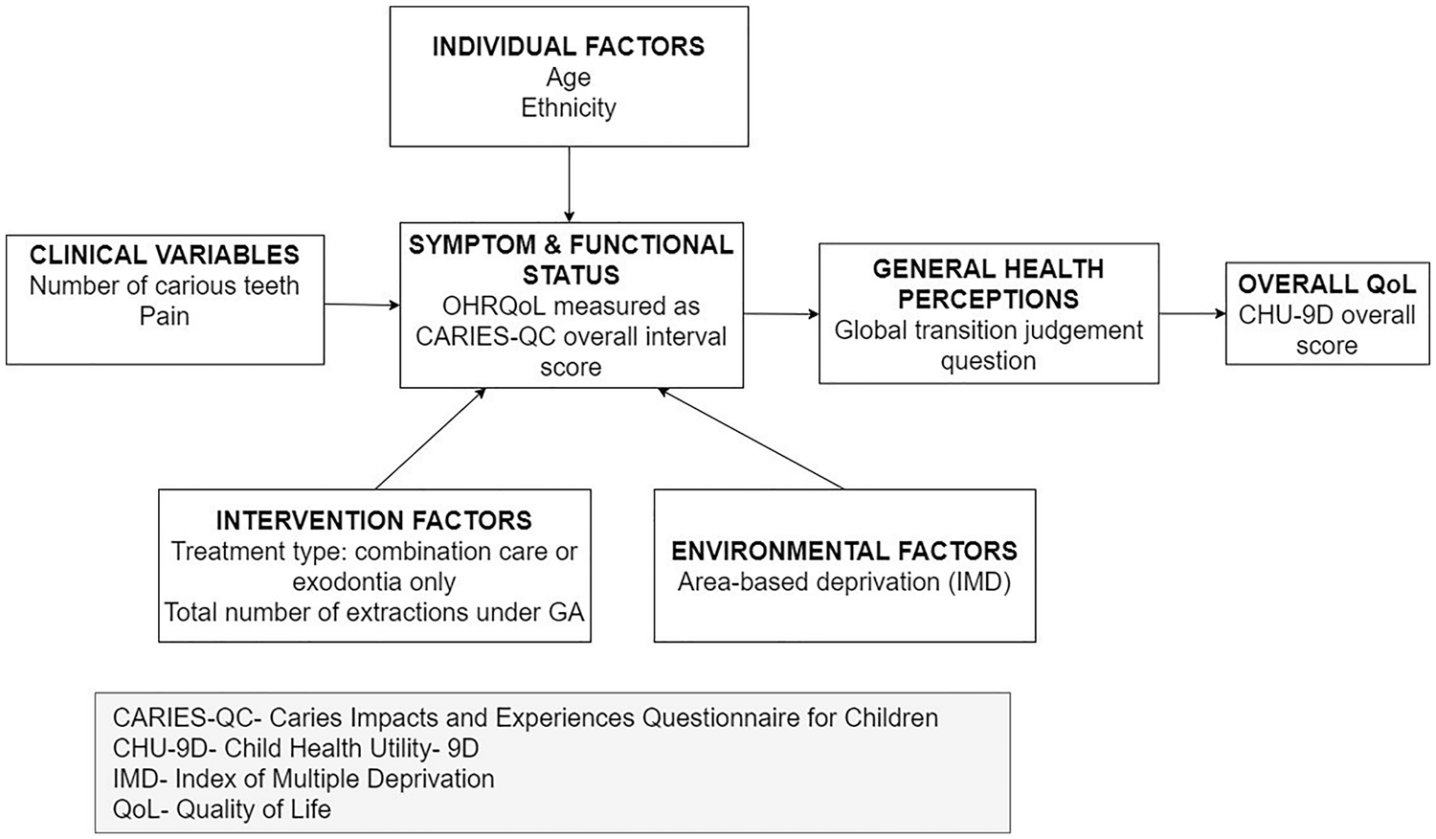
Proposed theoretical framework, adapted from the Wilson and Cleary model of HRQoL

A review of the literature allowed identification of the variables thought to be of greatest relevance to the present study, and these were included in the Wilson and Clearly model. An additional modification to the original model was the concurrent assessment of symptoms and function status, as a previous investigation suggested that children were less capable of making a distinction between these two aspects (Gilchrist et al. 2015). A validated caries-specific OHRQoL measure was thus used, the Caries Impacts and Experiences Questionnaire for Children (CARIES-QC), which concurrently measures child-reported symptoms and functions (Fig. 1).

### Individual factors

A record was made of the child’s age (years) as well as their ethnic group; categorised as ‘White British’ or ‘Black and Minority Ethnic’ (BAME).

### Clinical factors

The number of carious primary and permanent teeth, as a measure of disease experience, was obtained from the patients’ notes, based on the clinical and radiographic examination at their new patient appointment. A record was also made of any reported dental pain.

Treatment under GA was categorised as being solely extraction or a ‘combination’ approach. The combination care group included patients who had already received restorations in the clinic as part of their treatment plan, prior to progressing to GA extractions, as well as individuals who received both restorations and extractions under GA. Data were recorded for the total number of extractions and restorations (if applicable) performed under GA. Waiting times for treatment were also recorded for descriptive purposes.

### Environmental factors

The Index of Multiple Deprivation 2015 (IMD) score was used as an indicator of each participant’s deprivation status, which was derived from the child’s residential postcode. The IMD is a national measure of neighbourhood deprivation, used in England, based on key indicators, such as income, employment, and education status. Both IMD rank score and deprivation quintile were used for the purposes of this study.

### Quality of life outcomes

Quality of life was measured at two time points, first at the new patient assessment and then again 3-months following the dental GA. CARIES-QC was used to measure OHRQoL and the Child Health Utility 9D (CHU9D) to measure overall HRQoL (Stevens 2012; Gilchrist et al. 2018).

CARIES-QC was designed to be completed by children aged 5- to 16-years and is the only validated child-reported measure of OHRQoL specific to dental caries. It is purported to identify impacts related to caries which general OHRQoL measures may not be sensitive enough to detect (Gilchrist et al. 2014). The measure includes 12-items with a 3-point response format, where children rate whether they are affected ‘not at all’, ‘a bit’ or ‘a lot’, scoring 0, 1, 2, respectively. Total raw scores range from 0 to 24; the higher the score, the poorer the OHRQoL. To calculate change following treatment, to compare scores in the path analysis, raw scores are converted to the CARIES-QC interval scale score (CIS), which allows for the more accurate calculation of change at all points on the scale.

The CHU9D is a generic measure of HRQoL designed and validated for use with children aged 7- to 17-years (Foster Page et al. 2015). It has also been used in younger children with adult support. There is a growing literature to support its application in clinical research involving children with a wide range of medical conditions (Furber and Segal 2015). The CHU9D consists of 9-items, each with five ordinal responses (scored 1–5) that aim to assess the child’s functioning across domains such as worry, pain, tiredness, and daily routine. Overall scores can range from 9 to 45, where a higher score implies a greater impact on HRQoL (Stevens 2012).

### Data analysis

Data were entered and analysed using the Statistical Package for the Social Sciences ((IBM SPSS Statistics, version 24). Means and standard deviations were calculated for descriptive statistics. Total CIS and CHU9D scores at baseline and 3-months following treatment were determined and the Mann–Whitney test was used to determine change in scores. Change scores were calculated by subtracting follow-up scores from baseline scores for CARIES-QC and CHU9D, so a positive change score indicated improvement in OHRQoL and HRQoL, respectively. A negative change score represented a worsening of OHRQoL and HRQoL. Findings from statistical analysis were deemed significant if *p* < 0.05.

**Fig. 2:**
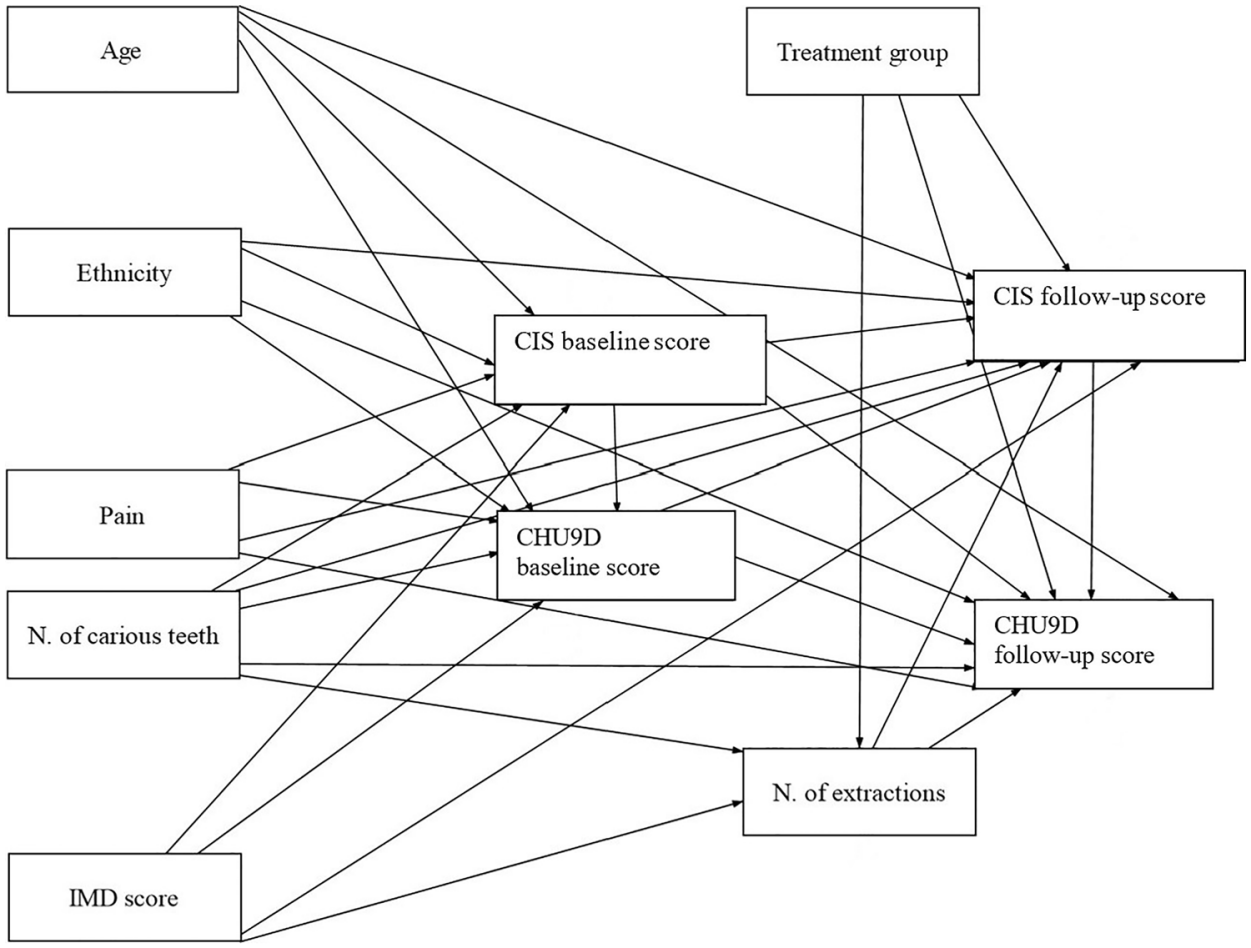
Full theoretical model, showing hypothesised paths between variables. Notes: N= Number. IMD= Index of Multiple Deprivation

Path analysis was conducted using STATA (StataCorp, version 15) to analyse the direct and indirect relationships between clinical, individual, and environmental factors and OHRQoL and identify predictors in the proposed model (Fig. 1). To carry out the analyses, the theoretical model was first converted into an ‘input’ path diagram (Fig. 2). Preliminary analysis revealed that data from some of the variables were not normally distributed thus Satorra–Bentler corrections were applied to all path analyses.

A parsimonious model was created by removing all non-significant paths, i.e., where *p* ≤ 0.1 (Olobatuyi 2006). The parsimonious model was compared to the full theoretical model using the Chi-square test for a difference to ensure there was no statistically significant difference between the two models. The standardised coefficients of each path in the resulting, parsimonious, were compared to identify which factors had greater effects within the model. The overall fit of the model was assessed using the following fit indices and thresholds: standardised root mean square residual (SRMR) ≤ 0.08, root mean square error of approximation (RMSEA) ≤ 0.06, Comparative Fit Index (CFI) ≥ 0.90, and Tucker Lewis Index (TCI) ≥ 0.90 (Hu and Bentler 1999).

## Results

### Sample characteristics

In total, 273 children were invited to take part in the study during a 2-year recruitment period (January 2017–January 2019). One hundred and six families declined study participation giving a response rate of 61.2%. Of those who did consent (*n* = 167), a further 82 were lost to follow up, leaving 85 parent–child dyads who completed the study (completion rate = 50.9%). Table 1 confirms that there were no fundamental differences between participants and non-participants were similar in terms of their demographic profiles.

**Table 1:** Comparison of participants’ demographic characteristics at baseline, of those followed up and those lost to follow-up

Variable	All (*n* = 167)	Followed up (*n* = 85)	Lost to follow-up (*n* = 82)	*p*-value
Age, years				
Range	5–14	5–11	5–14	0.14
Mean (± SD)	6.70 (± 1.69)	6.49 (± 1.53)	6.91 (± 1.83)	
Sex				
Male	79 (47.3%)	38 (44.7%)	41 (50.0%)	0.49
Female	88 (52.7%)	47 (55.3%)	41 (50.0%)	
Ethnicity				
White British	121 (72.5%)	62 (72.9%)	59 (72.0%)	0.89
BME	46 (27.5%)	23 (27.1%)	23 (28.0%)	
Deprivation (based on IMD score)				
Least deprived	10 (6.0%)	6 (7.1%)	4 (4.9%)	0.46
Less deprived	19 (11.4%)	10 (11.8%)	9 (11.0%)	
Average	16 (9.6%)	7 (8.2%)	9 (11.0%)	
More deprived	36 (21.5%)	14 (16.5%)	22 (26.8%)	
Most deprived	86 (51.5%)	48 (56.5%)	38 (46.3%)	
Safeguarding concern				
No	138 (82.6%)	77 (90.6%)	61 (74.4%)	0.19
Yes	20 (12.0%)	8 (9.4%)	12 (14.6%)	
Data missing	9 (5.4%)	0	9 (11.0%)	

Numbers, with percentages in brackets, are given unless otherwise stated

*SD* standard deviation, *BME* Black or minority ethnic group

*p*-values are for comparisons between the followed−up and lost to follow−up groups. As the data were not normally distributed, the Mann–Whitney *U* test was used to test for significant difference between the groups. Pearson’s chi−squared test was used to test for differences in categorical variables. There were no statistically significant results

The mean age of children who completed the study was 6.5 years (± 1.5, range 5–11). Around two-thirds (*n* = 62, 72.9%) of children were found to reside in the most deprived two-fifths of areas of England. Most children were White British (*n* = 62, 72.9%). The study group had a high caries experience with an average of 6.6 carious teeth (± 2.9; range 1–15). Pain was a presenting complaint for 70.6% (*n* = 60) of children.

In total, 72.9% (*n* = 62) of children underwent extraction only while 27.1% (*n* = 23) received combination care. The mean number of extractions in the extraction-only group was 6.7, compared to 5.1 in the combination care group, which was a statistically significant difference (*p* < 0.05). On average, children waited 9.4 (± 8.0) weeks for extractions only under GA which was a significantly shorter time than the 16.7 (± 9.6) weeks experienced by the combination care group (*p* = 0.001, Mann–Whitney *U* test).

Regarding quality-of-life outcomes, the mean CIS at baseline was 8.99 (± 4.29), with a range of 0–19.96. At 3-months follow-up, mean CIS was 4.55 (± 3.75) and scores ranged from 0 to 16.17. The mean change in CIS score was 4.47 (± 5.58), suggesting an improvement in OHRQoL following treatment. At baseline, mean CHU9D overall score was 13.58 (± 4.96) with a range of 9–31. At follow-up, the mean overall score was found to be reduced to 11.09 (± 3.07) with a range of 9–24. Overall, there was a mean reduction in CHU9D score of 2.48 (± 5.29), indicating improved HRQoL. Change in both CIS and CHU9D scores, between baseline and follow-up, were statistically significant (*p* < 0.001).

### Path analysis

The proposed theoretical model was a good fit for the data, meeting all four a priori criteria. The non-significant direct hypothesised paths were removed from this full theoretical model, which was re-estimated to obtain a statistically parsimonious model that was also a good fit for the data (Table 2).

**Table 2:** Summary of fit indices for full and parsimonious models

Model	*χ*^2^ (*df*, *p*-value)	RMSEA	CFI	TLI
Full theoretical model	6.88 (8, *p* = 0.55)*	< 0.001^a^	1.00^a^	1.04^a^
Parsimonious model	16.09 (28, *p* = 0.96)*	< 0.001^a^	1.00^a^	1.11^a^
Ideal value	*p* > 0.05	< 0.06	> 0.9	> 0.9

*χ*^*2*^ Chi-square test of model fit, *df* degrees of freedom, *RMSEA* Root Mean Square Error of Approximation, *CFI *Comparative Fit Index, *TLI* Tucker–Lewis Index

^a^Good model fit

There was no statistically significant difference between the full and parsimonious models (Chi-square, *p* = 0.97), suggesting the removal of the non-significant paths did not modify the original model. The parsimonious model was thus accepted and path analysis findings for this final model are given below.

Path analyses revealed that 47% (*R*^2^ = 0.465) and 25% (*R*^2^ = 0.249) of the variance in the CIS and CHU9D scores, respectively, 3 months post-treatment was accounted for by the variables in the model.

### Direct effects

**Fig. 3:**
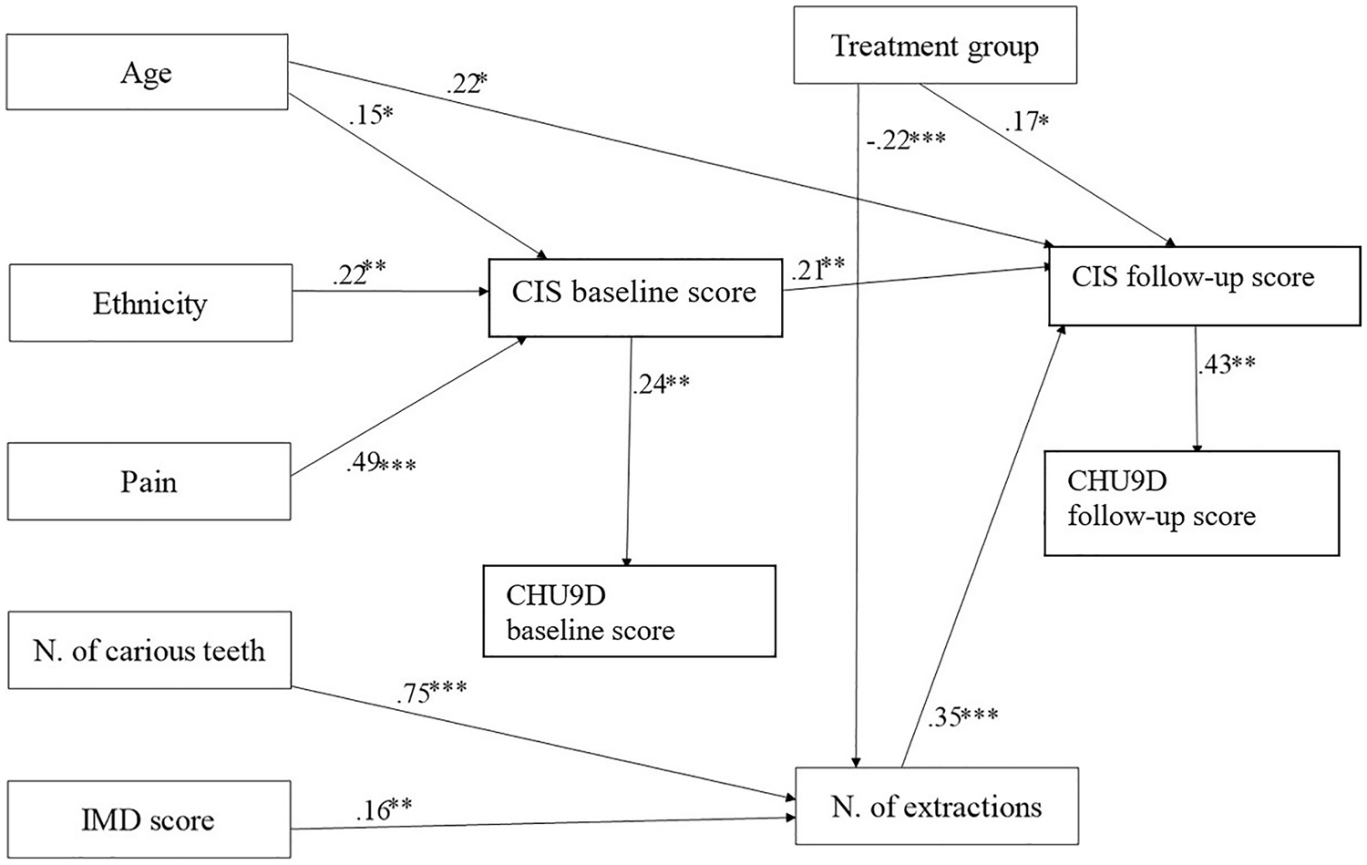
Statistically significant direct relationships in the final model, including standardized β- coefficients for each path. Notes: *p < 0.1, **p < 0.05, ***p < 0.01. N= Number. IMD= Index of Multiple Deprivation

Figure 3 shows the direct effects of the model. Ethnicity and pain both influenced child OHRQoL at baseline, but the pain had a greater effect (*β* = 0.49, *p* < 0.001) than ethnicity (*β* = 0.22, *p* = 0.02). A history of reported pain and belonging to a BAME group was associated with higher CIS scores i.e., worse OHRQoL at baseline. At follow-up, CIS scores were directly affected by the total number of extractions received and baseline CIS score. Increased numbers of extractions were associated with higher CIS scores at follow-up, or worse OHRQoL (*β* = 0.46, *p* < 0.001). Higher baseline CIS scores were associated with higher scores at follow-up but had less of an effect than the number of extractions (*β* = 0.18, *p* < 0.03). Higher CIS scores at baseline and follow-up were associated with worse overall HRQoL, i.e., higher CHU9D scores, at baseline (*β* = 0.28, *p* = 0.02) and follow-up (*β* = 0.36, *p* = 0.02), respectively. The total number of extractions carried out was most strongly affected by the number of carious teeth (*β* = 0.28, *p* = 0.02) and the treatment type (*β* = 0.28, *p* = 0.02), but the level of deprivation also had a significant effect (*β* = 0.03, *p* = 0.02). Increased numbers of extractions were associated with extraction-only treatment and higher levels of area-based deprivation.

### Indirect effects

**Fig. 4:**
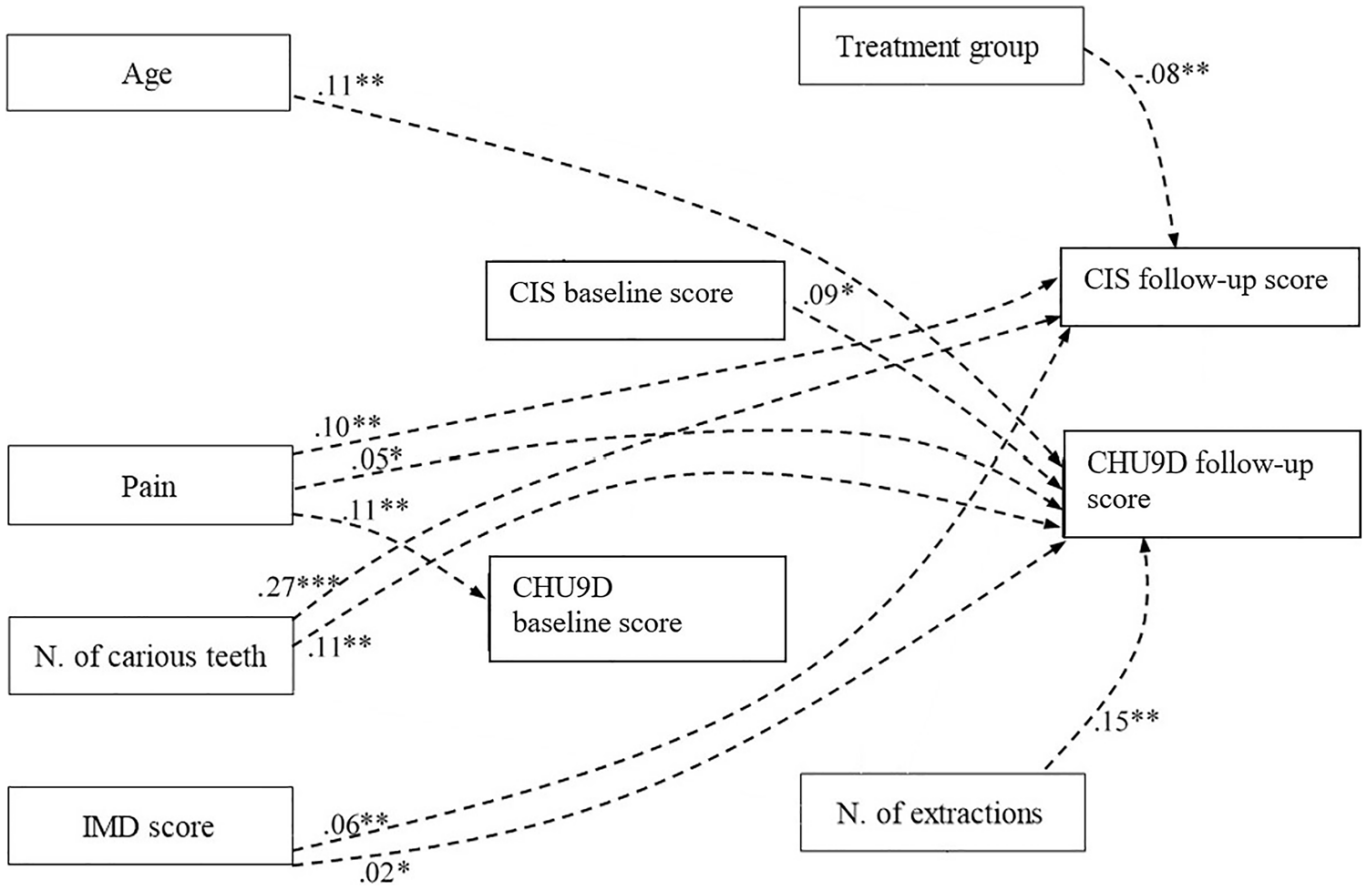
Statistically significant indirect relationships in the final model, including standardised β- coefficients for each path. Notes: *p < 0.1, ** *p* <0.05, ***p < 0.01. N= Number. IMD= Index of Multiple Deprivation

In addition, statistically significant indirect effects were also found within the model (Fig. 4). The number of carious teeth, treatment group, pain, and level of deprivation predicted the CIS at follow-up indirectly. The total number of extractions, the number of carious teeth and the age of the child all predicted CHU9D follow-up score indirectly. Number of carious teeth (*β* = 0.27, *p* < 0.001), treatment group (*β* = − 0.08, *p* = 0.005) and social deprivation (*β* = 0.06, *p* = 0.04) predicted OHRQoL at follow-up through number of extractions. OHRQoL at baseline mediated the link between pain and OHRQoL at follow-up (*β* = 0.10, *p* = 0.03) and pain and overall HRQoL (*β* = 0.12, *p* = 0.02). Number of extractions was indirectly linked overall HRQoL at follow-up via OHRQoL at follow-up (*β* = 0.15, *p* = 0.02), and via number of extractions and OHRQoL at follow-up (*β* = 0.11, *p* = 0.02).

## Discussion

This study was novel in that it sought to explore the direct and indirect effects of some key individual, environmental and clinical variables on children’s OHRQoL and HRQoL following treatment for dental caries under GA. The study benefitted from the use of child-completed validated measures for both HRQoL and caries-specific OHRQoL. Interestingly, although CHU9D has shown acceptable validity and reliability in a child population in New Zealand it was not found to be responsive to a change in caries status (Foster Page et al. 2014). The investigators, therefore, concluded that further research was warranted in child populations with higher caries experience, to determine if CHU9D was in fact responsive to change in caries levels. The children in the present clinical study had a particularly high overall caries experience, and CH9UD was indeed found to be responsive (to change) following caries management. It is also notable that the data were found to be a good fit to the proposed theoretical model, adapted from Wilson and Cleary.

This study also found that, overall, children’s OHRQoL and HRQoL are improved after caries management under GA, evidenced by a statistically significant reduction in OHRQoL and overall HRQoL scores. These findings are consistent with previous studies which have reported improved OHRQoL for children following treatment for dental caries under GA (Knapp et al. 2017).

However, in contrast to the findings of de Souza et al. (2017), who found no difference in the OHRQoL of children receiving combination care and those receiving extraction only GA, the present study identified that the treatment type did have a significant effect. This apparent disparity may relate to the use of a different measure of OHRQoL (P-CPQ) or inadequate sample size in the de Souza study. (de Souza et al. 2017). The path analysis in this research also allowed for control of other confounding factors which may have hidden an effect. Another key difference between the present study and the one undertaken by de Souza and colleagues relates to the treatment performed. The combination care group in this study included both children who received restorative treatment on clinic followed by extraction only under GA as well as those who had combination care under GA, whereas all restorative (i.e., combination) treatment was performed under GA.

Path analysis revealed that the type of treatment had a direct effect on children’s OHRQoL; patients who received combination care treatment reported poorer OHRQoL at follow-up than patients in the extraction only group. However, this effect was less than that seen for the number of extractions, meaning that children who had extraction only (and therefore had a higher number of teeth removed) were likely to have worse OHRQoL at follow-up. It is not possible to ascertain why the direct effect of combination care was worse OHRQoL at follow-up than for was the case for those who had extraction only. Further enquiry would be needed to explore differences between the groups in more detail to better understand this outcome. One explanation may be that children undergoing combined care were more likely to have additional needs, such as autism and/or attention deficit and hyperactivity disorder (ADHD), which necessitated a ward admission and pre-med. Thus, the whole experience of the dental GA may have been more distressing for these children. Indeed, it has recently been reported that conditions such as ADHD do impact children’s OHRQoL (Jamali et al. 2021). A further explanation may be the increased waiting time for combination care, which could have a negative impact. The waiting time for those undergoing extraction only in this study was 9.4 weeks, compared to a 16.7 week waiting list for children scheduled for combination care. The potential for long waiting times to negatively impact children’s wellbeing is well recognised. Goodwin et al. (2015) highlighted parental concerns about delayed hospital admissions for their child’s dental GA, including reports of ongoing pain and disturbed sleep It was not possible to consider waiting times as a separate variable in the present study, due to an inadequate sample size, thus this remains an important consideration for future work.

Several authors have reported that children living in more deprived areas are more likely to experience dental caries (Marcenes et al. 2013; Gilchrist et al. 2015). Findings from this study would concur, as the majority (72.9%) of participants lived in the most deprived areas of England. Concerningly, the present study also found that children living in more deprived areas also had a significantly higher number of extractions than those living in less deprived areas. Interestingly, path analysis failed to find a direct impact from deprivation on children’s OHRQoL and HRQoL. However, deprivation did have a statistically significant effect on the number of extractions received, and therefore indirectly impacted on both CIS and CHU9D scores at follow-up. Path analysis revealed that the level of deprivation was not impacting directly on OHRQoL and HRQoL in children. However, it was having a statistically significant effect on the number of extractions received, and therefore indirectly impacting on both CARIES-QC and CHU9D scores at follow-up.

As explanation for this difference in the number of extractions according to deprivation status remains speculative. Children who live in more deprived areas may be irregular dental attenders and their parents/carers may only take them to the dentist when they are already experiencing symptoms. In such cases, teeth may have a poorer prognosis in terms of restorative care and may require extraction. Another reason could be that some parents may prefer (primary) teeth to be extracted than restored, perceiving this to be a quicker and more reliable option. Furthermore, many parents may not understand the importance of primary teeth and place little value on them (Akhlaghi et al. 2017). Participants in this study (over a quarter) often had siblings who had also received dental treatment under GA, perhaps indicating that oral health advice, or the impact of a dental GA, had not brought about any significant behaviour change in that family. Indeed, it has been reported that some parents view a dental GA as an acceptable treatment approach, allowing their child to complete treatment in a single session (Anderson et al. 2004). Irrespective of these explanations, this study has highlighted the need for targeted oral health promotion programmes in areas of greatest deprivation to reduce inequalities in caries management for this vulnerable population.

In terms of individual variables, path analysis revealed that ethnicity and age impacted on children’s OHRQoL but had less impact than other factors. Older age and BAME status were associated with worse OHRQoL at baseline and follow-up, respectively. Ethnicity was only a significant factor at baseline, and age at follow-up. This finding, i.e., the effect was not present at both timepoints, is not easily explained. It may be there is an effect at both timepoints but it is too small to be captured by the sample size in this study. Other studies have proposed that poorer OHRQoL reported in some ethnic groups may not be solely related to ethnicity per se but is influenced by associated factors such as cultural differences in oral health practices, socioeconomic status, or parental education (Colak et al. 2013). Further work is therefore indicated to understand how factors related to ethnicity may impact on an individual’s OHRQoL. Regarding age, there are conflicting results in the literature, which perhaps explains why age only had a minor impact. It might be that increasing age results in increased awareness of oral problems, or more especially changes following treatment, and therefore age had an effect at follow-up but not at baseline.

An acknowledged limitation of this study was the sample size. The ideal statistical method for exploring the effects of variables would have been Structural Equation Modelling, which would allow the analysis of the outcomes as latent variables. However, due to expected difficulties with recruitment and retention in the study population, it was felt path analysis (which required a smaller sample size) was a suitable alternative. Other studies have employed path analysis to look at factors affecting quality of life outcomes using the Wilson and Cleary model (Ojelabi et al. 2017). This accepts the limitations of using OHRQoL and HRQoL as an observed variable, on the assumption that the measures are valid and reliable. The measures chosen were selected for this very reason: that they had previously shown good validity and reliability. A further point, which deserves reflection, is the fact that participants and their families were not assessed by a single clinician. Although all paediatric dentistry consultants (*n* = 8) in the host clinic have received standard training and adhere to national treatment-planning guidelines for caries management and dental GA, there will undoubtedly be some variability in individual communication styles and approaches to decision-making. It is acknowledged that some operator bias, conscious or unconscious, may therefore have come into play when formulating a treatment plan for an individual child with caries. This complex variable was not included in the analysis and therefore offers a fascinating area for future studies which seek to explore child- and parent-reported outcomes following interventions for caries management.

The high dropout rate, and therefore final sample size, limited the number of factors which could be considered in the path analysis. Several other factors, such as presence of a swelling, anterior caries or whether children had a safeguarding concern in place, were considered for inclusion but had to be removed due to the sample size requirements. The proposed theoretical model explained 52% of the variance in CARIES-QC scores, but only 24% of the variance in CHU9D scores. Further work is therefore needed to fully explain which factors impact on OHRQoL, and particularly HRQoL, in children who undergo a dental GA for the management of their caries.

In addition, the present study relied on the number of carious teeth as a measure of active disease, which gave no indication of the severity of symptoms. However, it was evident, from the path analysis, that reported pain was the most significant caries-related factor to impact on child-reported OHRQoL. This finding suggests that disease severity (i.e., likely pulpal involvement) may be a more important variable in predicting children’s OHRQoL than the number of carious teeth alone. It is interesting to reflect that most epidemiological studies use dmft/DMFT as an indicator of the burden of disease in populations, whereas the severity of disease in any one given tooth may present a greater impact than multiple asymptomatic carious teeth. Future studies could attempt to define caries severity and extent in more detail, for example using the ICDAS system, to better understand the effect of caries on OHRQoL.

## Conclusion

Despite some acknowledged limitations, this enquiry has demonstrated the value of using path analysis to identify which factors predict OHRQoL and HRQoL outcomes in children, following treatment of dental caries under GA. Notably, this study highlighted the potential for an increased number of extractions to have a negative impact on children’s QoL. Parents and children should be fully informed about the potential risks of choosing to extract multiple primary teeth where there is the possibility of restoring them.

## Data Availability

The data sets used and analysed during the current study are available from the corresponding author on reasonable request.
